# Sex‐Dependent Effects of CSF1R‐Mediated Myeloid Cell Depletion in a Mouse Model of Multiple System Atrophy

**DOI:** 10.1111/ejn.70586

**Published:** 2026-06-18

**Authors:** Kristina Battis, Michael Mante, Isabel Naumann, Marie Andert, Ha Yeon Kim, Wei Xiang, Robert A. Rissman, Jürgen Winkler, Alana Hoffmann

**Affiliations:** ^1^ Department of Molecular Neurology University Hospital Erlangen, Friedrich‐Alexander‐Universität Erlangen‐Nürnberg Erlangen Germany; ^2^ Department of Physiology and Neuroscience Keck School of Medicine of the University of Southern California San Diego California USA; ^3^ Biotech Research and Innovation Center University of Copenhagen Copenhagen Denmark; ^4^ Center for Rare Disorders (ZSEER) University Hospital Erlangen, Friedrich‐Alexander Universität Erlangen‐Nürnberg (FAU) Erlangen Germany; ^5^ Keenan Research Centre for Biomedical Science and Barlo Multiple Sclerosis Centre St. Michael's Hospital Toronto Ontario Canada; ^6^ Department of Immunology The University of Toronto Toronto Ontario Canada; ^7^ UK Dementia Research Institute, Centre for Discovery Brain Sciences, Chancellor's Building University of Edinburgh Edinburgh UK

**Keywords:** colony‐stimulating factor 1 receptor, multiple system atrophy, myeloid cells, sex differences

## Abstract

Sex‐dependent differences in neurodegenerative disorders are becoming increasingly relevant in diagnosis and development of therapeutic targets. Although multiple system atrophy (MSA), a devastating and rapidly progressing atypical parkinsonian disorder, is distributed equally among sexes, sex‐specific differences have been reported regarding autonomic dysfunctions, severity of motor symptoms, and survival rate, suggesting a need for sex‐specific treatment approaches. Neuroinflammation is a prominent hallmark in MSA pathology. Distinct activation patterns and increased phagocytosis of central nervous system (CNS) myeloid cells were observed, indicating a damaging role in this disease. In our previous study, we showed that colony‐stimulating factor 1 receptor (CSF1R)–mediated depletion of myeloid cells in a mouse model of MSA led to a two‐faced outcome, comprised of a prolonged survival and delayed onset of neurological dystonia‐like symptoms, but also an aggravated motor phenotype and loss of dopaminergic neurons. Here, we re‐analyzed our previous findings to study sex‐specific effects in MSA in the presence and absence of CNS myeloid cells. Intriguingly, myeloid cell depletion was initially more effective in male animals. Although no sex‐specific effects were detected in motor behavior, the occurrence of dystonia‐like neurological symptoms was reduced, and neuronal loss was alleviated in male animals. Together, our findings provide insight into sex‐specific differences of CSF1R‐mediated myeloid cell depletion in MSA, emphasizing the need to study sex‐specific treatment strategies in neurodegenerative disorders.

## Graphical abstract

Myeloid cell depletion in males is rapid and initially more effective than in females, which leads to reduced occurrence of dystonia‐like neurological symptoms and alleviated neuronal loss in a mouse model of MSA. Overall, these findings indicate that the therapeutic efficacy of CSF1R‐mediated myeloid cell depletion in MSA is sex‐specific.

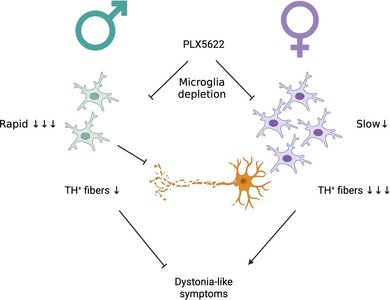

**Abbreviations**
CNS: central nervous system
CON: control
CSF1R: colony‐stimulating factor 1 receptor
DOB: date of birth
IBA1: ionized calcium‐binding adapter molecule 1
MSA: multiple system atrophy
PLX: PLX5622
SNpc: substantia nigra pars compacta
TH: tyrosine hydroxylase
WT: wildtype
α‐syn: α‐synuclein

## 1 Introduction

Sex is a significant biological variable that affects the prevalence and incidence of neurological disorders (Gillies and McArthur 2010; Loke et al. 2015; McCarthy et al. 2012; Young and Pfaff 2014). For instance, Alzheimer's disease and Parkinson's disease, the two most prevalent neurodegenerative disorders, are distributed unevenly across sexes (Mielke et al. 2014; Patel and Kompoliti 2023). In addition, sex differences in frequency, pathology, and clinical representation are observed in multiple sclerosis patients (Li et al. 2017; Ryan and Mills 2022). One important contributing factor may be the sex difference in both innate and adaptive immune responses that is evolutionarily conserved across diverse species including humans, mice, birds, and fish (Klein and Flanagan 2016; Ryan and Mills 2022).

A severe, white matter‐specific immune response of myeloid cells, predominantly microglia, is present in multiple system atrophy (MSA), a rare atypical parkinsonian disorder (Hoffmann et al. 2019; Ishizawa et al. 2004). MSA is characterized by the accumulation of α‐synuclein (α‐syn) within oligodendroglial cytoplasmic inclusions (Dickson et al. 1999; Papp et al. 1989), leading to oligodendrocyte dysfunction and consequently to a profound myelin deficit (Ettle et al. 2016; Ettle et al. 2014; Matsuo et al. 1998; Meszaros et al. 2021). Although MSA is distributed equally across sexes (Ben‐Shlomo et al. 1997), differences in the clinical characteristics vary between men and women. In particular, men are more likely to suffer from orthostatic intolerance, whereas a longer survival rate from diagnosis with early manifestation and more severe motor symptoms was detected in women (Bailey et al. 2023; Coon et al. 2019; Cuoco et al. 2020; Yamamoto et al. 2014). Thus, we propose that sex needs to be considered as an important variable when studying MSA pathology and developing therapeutic strategies.

In a recent study, we used the colony‐stimulating factor 1 receptor (CSF1R) inhibitor PLX5622 to deplete myeloid cells in a mouse model for MSA, which expresses human α‐syn under the control of an oligodendrocyte‐specific myelin basic protein (MBP) promoter (MBP29‐hα‐syn mice) (Battis et al. 2022). Neuropathology of these mice is characterized by myelin deficit, neuronal dysfunction, and predominant white matter‐associated neuroinflammatory responses. Long‐term PLX5622 administration profoundly reduced myeloid cell numbers in MBP29‐hα‐syn mice and had a two‐faced effect, comprised of a prolonged survival and delayed onset of neurological symptoms, but accompanied by an aggravation of motor symptoms and neuronal and synaptic alterations (Battis et al. 2022). However, in our previous study, we did not focus on potential sex‐specific effects of PLX5622 administration.

Intriguingly, significant sex differences upon CSF1R‐mediated myeloid cell depletion were already observed previously in wildtype (WT) Sprague Dawley rats (Sharon et al. 2022) and in the neurodegenerative mouse model neuronal ceroid lipofuscinoses (CLN diseases) (Berve et al. 2020). Depletion of microglia using PLX5622 in embryonic mice resulted in sex‐specific behavioral effects, including anxiolytic‐like behavior and long‐term hyperactivity in females (Rosin et al. 2018). Furthermore, CSF1R inhibition using PLX3397 led to increased depletion efficiency and upregulation of different signaling pathways upon depletion in male compared with female C57BL6/J mice (Le et al. 2025). The data indicate that CNS myeloid cells and their function may differ between males and females. Here, we reanalyzed the data obtained by Battis et al. (2022), discriminating between sexes to analyze sex‐specific differences in an MSA mouse model in the presence and absence of myeloid cells. We focus on behavioral and structural analyses in order to study whether depletion of myeloid cells has distinct effects in males versus females.

## 2 Materials and Methods

### 2.1 Mice

Heterozygous MBP29‐hα‐syn mice and non‐transgenic WT littermates (hybrid background strain: B6D2F1) were maintained under standard animal housing conditions with a 12‐h dark–light cycle and access to food and water ad libitum (Gow et al. 1992; Shults et al. 2005). Data acquired from the identical cohort of mice as presented in Battis et al. (2022) were reanalyzed due to ethical reasons, implementing the “3Rs” in animal research (i.e., Reduction, Refinement, and Replacement). All rodent experiments complied with NIH (National Institute of Health) guidelines for good animal care and use, and all procedures were reviewed and approved by the University of California, San Diego Institutional Animal Care and Use Committee. The numbers of animals are summarized in Table 1.

**TABLE 1:** Number of animals used for the analysis. Animal numbers were equally distributed across PLX5622‐ and control‐administered animals if not indicated otherwise.

Experiment	WT (CON/PLX5622)		MBP29 (CON/PLX5622)	
	Female	Male	Female	Male
Motor behavior/neurological symptoms	15 (CON)	16 (CON)	15 (CON)	11 (CON)
	10 (PLX5622)	13 (PLX5622)	13 (PLX5622)	10 (PLX5622)
IBA1 (3 weeks)	3	2	3	2
IBA1 (3 months)	5	5	5	5
PU.1	3	3	3	3
TH	5	5	5	5

### 2.2 PLX5622 Administration

As described previously (Battis et al. 2022), PLX5622 was provided by Plexxikon (CA, USA). The compound was formulated at 1200 mg/kg in AIN‐76A standard chow provided by Research Diets Inc. (NJ, USA). Mice were fed with PLX5622‐containing or control standard AIN‐76A chow beginning at 4 weeks of age for a period of 3 or 12 weeks.

### 2.3 Behavioral Analysis

All behavioral analysis was performed as previously described (Battis et al. 2022).

#### 2.3.1 Beam Walking Test

Animals were trained three times using a horizontal round beam (1 m × 2.5 cm) prior to three test trials. During testing, animals traversed along a smaller horizontal round beam (1 m × 1.3 cm). A trial was completed when the animal reached the platform at the end of the beam or 1 min expired. The number of foot slips, total forward distance, and time traversing the beam were recorded.

#### 2.3.2 Pole Test

Animals were placed facing upward on a vertical wooden pole (50 cm × 1 cm), and the time to climb down (total time) was recorded. Total descent time was further divided into time to turn around and time to descend head downward. A trial was completed when the animal placed all four paws on the ground or after 2 min. Mice performed three training trials and five test trials on the same day, with 2‐min rest periods between trials.

#### 2.3.3 RotaRod Analysis

Animals were placed on a horizontal rotating rod accelerating from 0 to 40 rpm over 4 min, and the latency to fall was recorded. The animals performed five training trials at accelerating speeds of 10, 20, and 40 rpm 1 day prior to the testing. Seven test trials were conducted, and the latency to fall was measured for each trial.

#### 2.3.4 Scoring of Dystonia‐Like Behavior

Starting at 10–12 weeks of age, MBP29‐hα‐syn mice display brief, sporadic dystonic episodes in response to sensory stimuli or movements (e.g., animal handling) followed by full recovery within seconds. Dystonia‐like behavior was scored weekly from 6 weeks of treatment (10 weeks of age) based on the presence or absence of the episodes upon cage opening and animal handling.

### 2.4 Tissue Processing

For histological analysis, animals were perfused with 0.9% NaCl solution, and one hemisphere was postfixed in 4% paraformaldehyde overnight, dehydrated in 30% sucrose, and coronally sectioned (40 μm).

### 2.5 Histology

For immunohistochemistry staining, antigen retrieval was performed with citrate buffer (0.1 M) for 30 min at 80°C, intrinsic peroxidase was blocked by incubation in 0.6% H_2_O_2_ for 60 min, and unspecific antibody‐binding was blocked using 0.3% Triton‐X100 and 3% donkey serum for 1 h. Rabbit anti‐IBA1 (WAKO 019‐19741; 1:500) was incubated in blocking solution overnight at 4°C. Donkey anti‐rabbit‐Biotin (Dianova 711‐065‐152; AB_2340593, 1:1000) was incubated at room temperature for 1 h in blocking solution. Afterwards, avidin–biotin–peroxidase complex (Vectastain Elite, Vector Laboratories) was added for 1 h, followed by peroxidase detection (25 mg/mL diaminobenzidine (DAB), 0.01% H_2_O_2_ in TBS). Sections were mounted on glass slides using NeoMount (Merck).

For immunofluorescence staining, sections were treated with citrate buffer and blocking solution as described above. The following primary antibodies were incubated in blocking solution overnight at 4°C: Rabbit anti‐PU.1 (Cell Signaling 2266S; AB_10692379; 1:400) or rabbit anti‐TH (Millipore AB152; AB_390204; 1:500). Donkey anti‐rabbit‐Alexa647 (Dianova 711‐605‐152; AB_2492288; 1:1000) was added for 1 h at room temperature. Nuclei were counterstained by incubation with 4′,6‐diamidino‐2‐phenylindole (DAPI) at room temperature for 10 min (Sigma, 1:10,000). Sections were mounted on glass slides and covered with Prolong Gold (Invitrogen).

### 2.6 Data Analysis

The animal numbers of the acquired data from Battis et al. (2022) are indicated in Table 1. A minimum of 10 animals per treatment and sex were used for behavioral assessment. During the treatment period of 12 weeks, eight control‐treated MBP29‐hα‐syn mice died (four females and four males). Structural analysis was conducted using three to five animals per group and sex (see Table 1).

Investigators were blinded throughout all experiments including behavioral and image analysis. Of note, limitations to blinding have to be mentioned due to the different coloring of the PLX5622 and control diet. In addition, microglia numbers and morphologies change significantly in transgenic animals as well as in PLX5622‐treated animals, so blinding of investigators may have been compromised by these factors.

### 2.7 Statistics

Statistical analyses and visualization were performed using GraphPad Prism 9 software (version 9, GraphPad Software, USA). Normal distribution was assessed by the Shapiro–Wilk normality test. For normally distributed data, two‐way ANOVA in combination with Sidak's multiple comparison post hoc test was used, and the Kruskal–Wallis test in combination with Dunn–Bonferroni post hoc method for not normally distributed data. Significant sex difference is indicated with * throughout the manuscript. # is used to indicate a significant difference related to treatment, genotype, or overall difference of all groups.

## 3 Results

### 3.1 PLX5622 Depletes Myeloid Cells Differently in Males and Females

**FIGURE 1:**
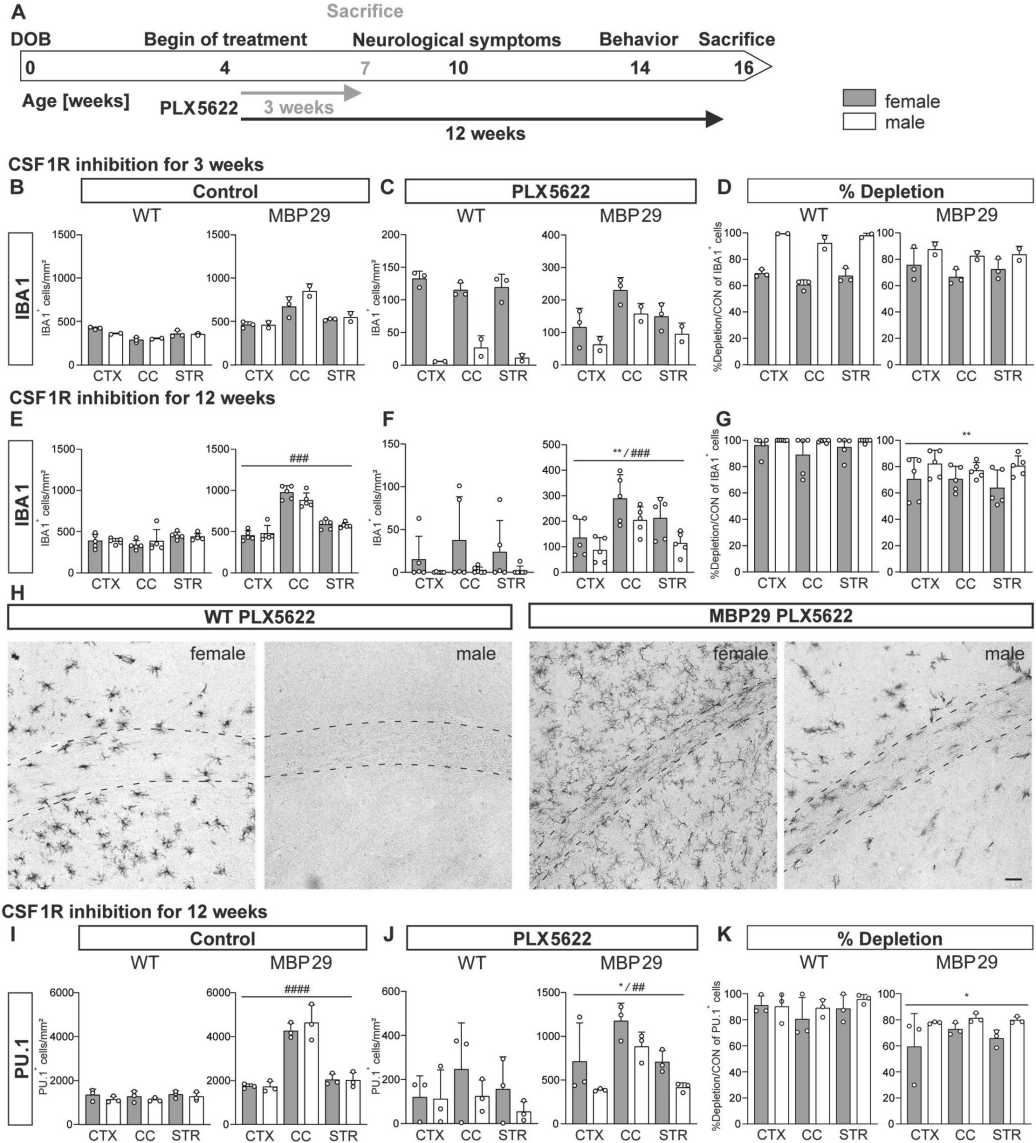
Sex‐dependent depletion of myeloid cells upon PLX5622 administration. (A) Experimental paradigm of PLX5622 (1200 mg/kg) administration of WT and MBP29‐hα‐syn mice for 3 and 12 weeks (DOB: date of birth). Quantification of IBA1^+^ cell density in the cortex (CTX), corpus callosum (CC), and striatum (STR) of WT and MBP29‐hα‐syn mice fed with control (B, 3 weeks; E, 12 weeks) or PLX5622 diet (C, 3 weeks; F, 12 weeks) and percentage of PLX5622‐mediated myeloid cell depletion (D, 3 weeks; G, 12 weeks). (H) Representative images of IBA1^+^ cells in female and male WT and MBP29‐hα‐syn mice receiving PLX5622 diet for 12 weeks. Scale bar: 50 μm. Quantification of myeloid cells expressing the transcription factor PU.1 in respective brain regions of WT and MBP29‐hα‐syn mice fed with control (I) or PLX5622‐diet (J) for 12 weeks and percentage of myeloid cell depletion (K). Data represent mean + SD (*n* = 2–3 animals per group for short‐term and *n* = 3–5 for long‐term administration). Statistical analyses were performed using Shapiro–Wilk to test for normal distribution, followed by Kruskal–Wallis (E, I, WT: F, G, J, K) or two‐way ANOVA (MBP29‐hα‐syn: F, G, J, K). # = regional difference; * = sex difference. #/*p ≤ 0.05; ##/**p ≤ 0.01; ###/***p ≤ 0.001; ####/****p ≤ 0.0001. Bar graphs indicate females (gray) and males (white).

To analyze the efficacy of myeloid cell depletion, as well as the tolerability of PLX5622 in males and females, MBP29‐hα‐syn and WT mice were fed with 1200 mg/kg PLX5622‐containing chow for a period of 3 or 12 weeks (Figure 1A). Administration was started at 4 weeks of age due to the early myeloid cell activation in the MBP29‐hα‐syn model observed already at postnatal day 21 (Hoffmann et al. 2019). We focused on distinct brain regions displaying severe (corpus callosum, striatum) or less severe (cortex) neuroinflammation (Hoffmann et al. 2019) and quantified the number of ionized calcium–binding adapter molecule 1 (IBA1)^+^ cells as shown by Battis et al. (2022).

Notably, no sex differences in the number of IBA1^+^ cells were detected in the cortex, the corpus callosum, or the striatum of MBP29‐hα‐syn and WT mice receiving a control diet after short‐term (Figure 1B) and long‐term administration (Figure 1E). Indeed, PLX5622 was well tolerated in both male and female animals, and depletion efficacy of IBA1^+^ myeloid cells was indicated to be more efficient in WT mice as described before (Battis et al. 2022) (Figure 1C,F). An initial small‐scale experiment with only two male and three female mice suggested that depletion efficiency may be more pronounced in male compared with female animals after 3 weeks of PLX5622 administration (Figure 1B–D). Because of the small number of animals receiving PLX5622 or control diet for 3 weeks (*n* = 2–3), we were unable to perform statistical analysis for this pilot study. However, power calculation using openepi.com revealed that *n* = 1 would be sufficient for detecting differences between sexes in each brain region (cortex, corpus callosum, striatum) of WT animals receiving PLX5622, suggesting a potential biological relevance (Table 2).

**TABLE 2:** Power calculation analysis. Power calculation was performed using https://www.openepi.com/SampleSize/SSMean.htm to determine the minimally required sample size for a significant and biologically relevant effect for comparing males and females in each group and brain region (confidence interval [two‐sided]: 95%; statistical power: 80%, ratio of sample size: 1).

		CTX			CC			STR		
		Female	Male	Required sample size	Female	Male	Required sample size	Female	Male	Required sample size
WT PLX	Mean	132.49	5.72	1	115.11	26.54	1	119.25	10.86	1
	SD	11.80	0.30		11.18	18.61		20.15	6.56	
WT CON	Mean	422.14	358.94	1	287.32	302.74	32	358.19	351.49	303
	SD	14.34	8.52		30.28	6.94		38.47	15.71	
% Change WT	Mean	68.61	98.41	1	59.94	91.23	1	66.71	96.91	1
	SD	2.80	0.08		3.89	6.15		5.62	1.87	
MBP29 PLX	Mean	116.50	62.50	11	229.90	157.06	4	149.30	95.28	8
	SD	57.86	25.92		39.70	32.46		41.29	33.74	
MBP29 CON	Mean	461.27	458.81	4553	669.97	848.80	5	524.10	546.53	73
	SD	35.81	47.26		113.65	83.88		10.85	67.31	
% Change MBP29	Mean	74.74	86.38	11	65.68	81.50	2	71.51	82.57	7
	SD	12.54	5.65		5.93	3.82		7.88	6.17	

To analyze sex‐specific differences in more detail, quantification of IBA1^+^ cells (*n* = 5; Figure 1E–H) was performed after 12 weeks of PLX5622 administration, which revealed a widespread depletion of myeloid cells in both sexes in WT and MBP29‐hα‐syn mice. In comparison with short‐term treatment, long‐term depletion of myeloid cells over 12 weeks efficiently reduced the number of IBA1^+^ cells in all analyzed brain regions of WT animals (Figure 1F). Although numbers were significantly reduced in both sexes compared with 3 weeks of PLX5622 treatment, some WT female animals still showed residual myeloid cells in all brain regions. Similar results were obtained by quantification of the myeloid cell transcription factor PU.1, showing a significant reduction of myeloid cell nuclei in both sexes but an increased number of residual nuclei in females (*n* = 3; Figure 1I–K). Significant differences were detected between males and females in MBP29‐hα‐syn mice, with more pronounced depletion efficiency of IBA1^+^ cells and PU.1^+^ nuclei in males (Figure 1F,G,J,K).

In conclusion, sex‐specific differences in myeloid cell depletion may be detected as early as 3 weeks of PLX5622 treatment, both in WT and MBP29‐hα‐syn animals. Particularly, myeloid cell depletion in male mice appeared to be more efficient already after 3 weeks of PLX5622 administration, further suggesting that female myeloid cells might take longer to respond or may be more resilient toward PLX5622 treatment. In addition, long‐term administration of PLX5622 reveals that some myeloid cells show resistance toward complete depletion, which was more prominent in females (68.2% in MBP29‐hα‐syn and 93.2% in WT depleted) compared with males (79.7% in MBP29‐hα‐syn and 99.6% in WT depleted). Overall, male and female animals might have different depletion dynamics independent of the genotype, with male myeloid cells already reaching their maximum depletion rate after 3 weeks of PLX5622 administration, whereas female myeloid cells take longer.

### 3.2 Males Perform Worse in RotaRod Test Independent of Myeloid Cell Depletion

**FIGURE 2:**
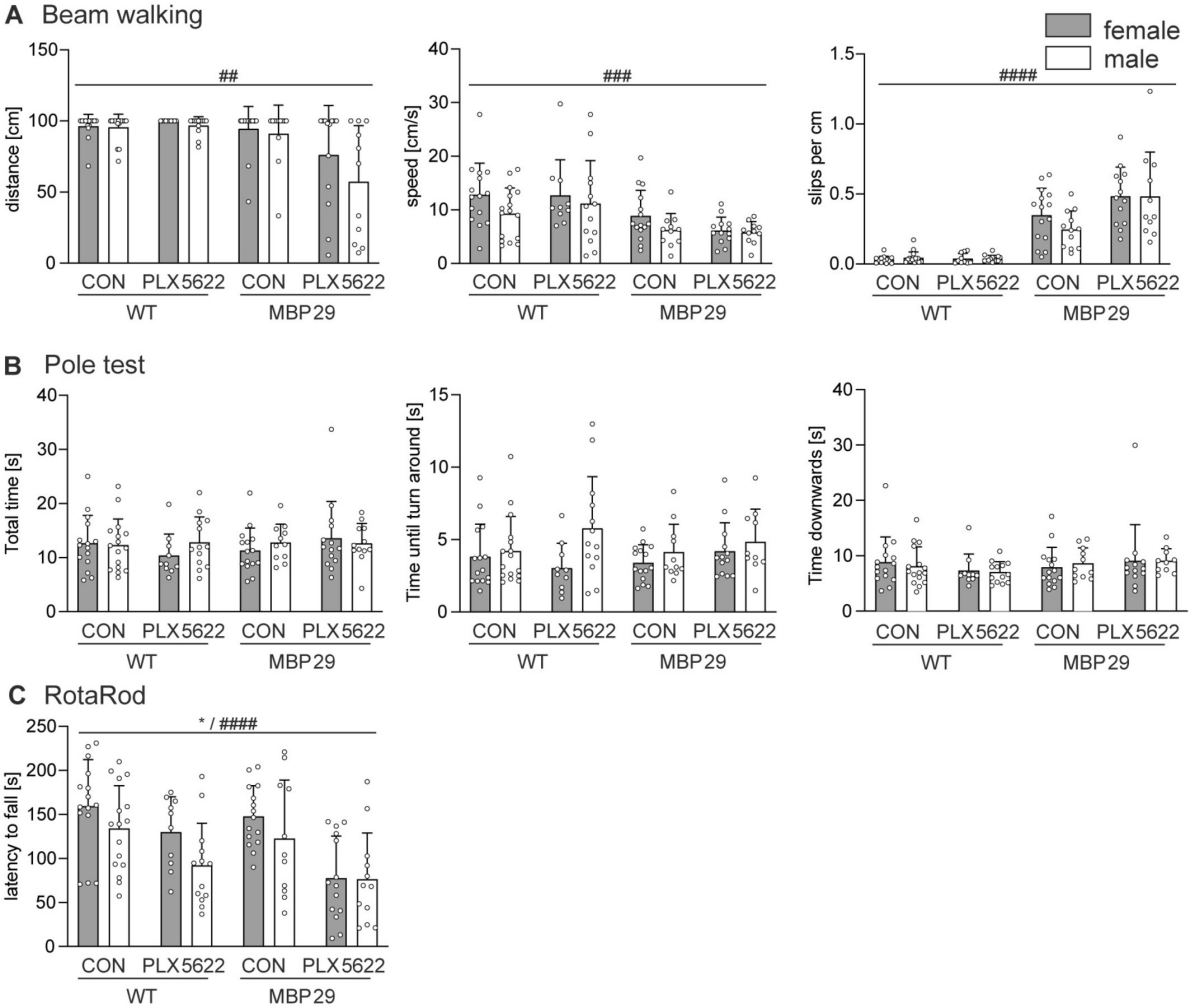
Overall sex‐ and treatment‐specific differences in RotaRod test. PLX5622 (1200 mg/kg) administration of MBP29‐hα‐syn and WT mice was performed for 12 weeks. Seven to 12 days prior to sacrifice, behavioral testing was performed including (A) beam walking, (B) pole, and (C) RotaRod test. Statistical analyses were performed using Shapiro–Wilk to test for normal distribution and Kruskal–Wallis (A, B) or two‐way ANOVA (C). # = overall differences between groups or treatment difference (C); * = sex difference. #/*p ≤ 0.05; ##/**p ≤ 0.01; ###/***p ≤ 0.001; ####/****p ≤ 0.0001.

In our previous study, we demonstrated an aggravation of the motor phenotype of MBP29‐hα‐syn mice upon CSF1R inhibition for 12 weeks. To assess the functional consequences of myeloid cell depletion in a sex‐dependent manner, we reanalyzed the behavioral data separated by sex. As described in Battis et al. (2022), we identified differences related to genotype and treatment, with MBP29‐hα‐syn mice receiving PLX5622 performing worse in the beam walking (Figure 2A) and RotaRod test (Figure 2C). In general, no differences were observed between female and male MBP29‐hα‐syn mice in their motor behavior, including distance travelled along the beam, speed, and slips per centimeter (Figure 2A) in the beam walking test. Similarly, no differences between sexes, treatment, or genotype were observed in the pole test (total time needed to climb the pole, time until turnaround, and time climbing downward) (Figure 2B). Interestingly, performance in the RotaRod test was dependent on treatment and sex (without interaction), with males having an overall lower latency to fall off the rod than females (Figure 2C). Correlating the latency to fall with the weight of the animals, we identified a negative correlation only in WT animals treated with PLX5622. However, no significant correlation with the latency to fall was detected in WT mice treated with control diet, or in MBP29‐hα‐syn mice, regardless of whether they were treated with control diet or PLX5622 (data not shown).

Taken together, there was no sex‐dependent effect of long‐term administration of PLX5622 in the motor phenotype of MBP29‐hα‐syn mice; however, males overall showed a lower latency to fall off the RotaRod, which was independent of genotype and treatment.

### 3.3 PLX5622 Administration Modifies Dystonia‐Like Behavior in MBP29‐hα‐syn Mice in a Sex‐Dependent Manner

**FIGURE 3:**
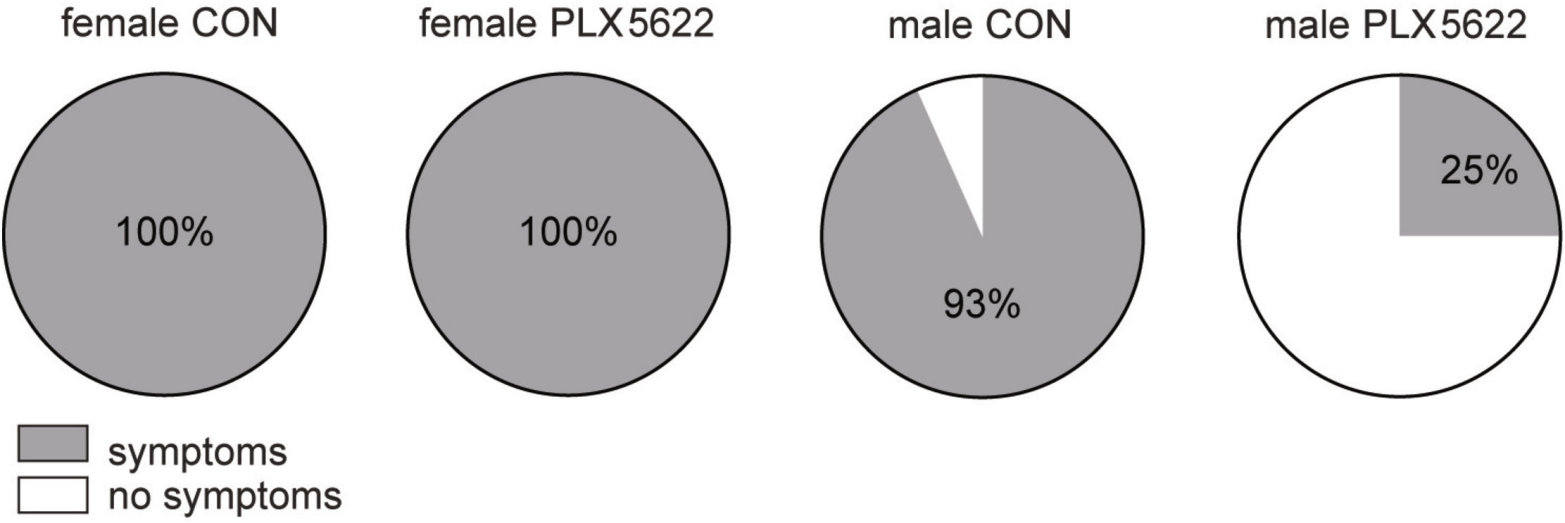
Dystonia‐like behavior upon PLX5622 administration. Quantification of male and female MBP29‐hα‐syn mice fed with control or PLX5622 diet for 12 weeks (*n* = 12–19) developing dystonia‐like neurological symptoms. Presence of dystonic symptoms (gray) was analyzed weekly beginning at 10 weeks of age until sacrifice at 16 weeks.

In contrast to worsened motor symptoms, we showed that long‐term administration of PLX5622 ameliorated stress‐induced dystonia‐like behavior in MBP29‐hα‐syn mice (Battis et al. 2022). To our surprise, separating by sex, we identified that the frequency of dystonia‐like behavior was mainly reduced in males. Only 25% (3/12) of male animals, but all females, developed dystonia‐like behavior upon PLX5622 treatment over time until the day of sacrifice. In comparison, 93% (14/15) of males and all females in the control‐treated cohort developed dystonia‐like symptoms (Figure 3). These data indicate that early myeloid cell depletion may improve dystonia‐like behavior, particularly in males.

### 3.4 Male MBP29‐hα‐syn Mice Show Increased TH^+^ Fiber Density in the Striatum Upon PLX5622 Administration

**FIGURE 4:**
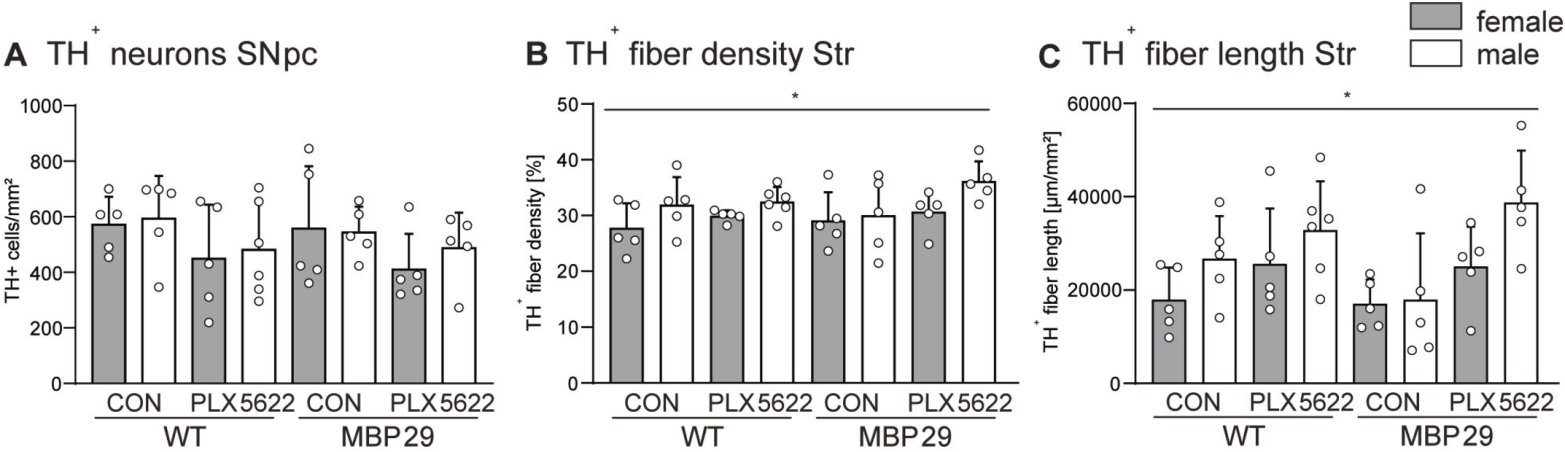
Sex‐dependent neuronal phenotype upon PLX5622 administration. Quantification of (A) TH^+^ cells in the substantia nigra pars compacta (SNpc), (B) TH^+^ fiber density, and (C) length in the striatum (Str) (*n* = 5). Data represent mean + SD. Statistical analyses were performed using Shapiro–Wilk to test normal distribution and Kruskal–Wallis (A) or two‐way ANOVA (B, C). * = sex. *p ≤ 0.05.

Since PLX5622 treatment affected motor symptoms and dystonia‐like behavior of MBP29‐hα‐syn mice in our previous study, we investigated the integrity of the dopaminergic striato‐nigral system upon PLX5622 administration. Re‐analysis of tyrosine hydroxylase (TH)^+^ neuronal density in the substantia nigra pars compacta revealed no difference between male and female animals receiving PLX5622 or control diet (Figure 4A). Interestingly, the overall TH^+^ fiber density in the striatum was higher in males independent of treatment and genotype (Figure 4B), and TH^+^ fiber length was increased in PLX5622‐treated males independent of the genotype (Figure 4C), indicating that early augmented myeloid cell depletion in male MBP29‐hα‐syn mice may increase fiber density in the striatum compared with female animals.

## 4 Discussion

Sex is a significant variable in the prevalence and pathology of neurological disorders and has been a focus of interest in an increasing number of studies in recent years (Piscopo et al. 2021). Although MSA is distributed equally across sexes (Ben‐Shlomo et al. 1997), some sex‐specific differences have been reported. For instance, female patients showed more severe motor symptoms compared with male patients at similar age and disease duration (Coon et al. 2019; Cuoco et al. 2020). Moreover, a longer survival rate from diagnosis with an early manifestation of motor deficits was observed in female patients, whereas male patients were more likely to suffer from urinary symptoms (Bailey et al. 2023; Coon et al. 2019; Yamamoto et al. 2014). Thus, sex‐specific treatment of MSA is an important consideration in improving the quality of life and disease progression in individual MSA patients.

Pharmacological inhibition of CSF1R has been studied in recent years as a potential therapeutic target in different neurodegenerative diseases, including Alzheimer's disease (Dagher et al. 2015; Spangenberg et al. 2019), multiple sclerosis (Nissen et al. 2018), Parkinson's disease (Li et al. 2021; Thi Lai et al. 2024), and MSA (Battis et al. 2022; Gauer et al. 2024). Similar to other mouse models, CSF1R inhibition in MBP29‐hα‐syn mice resulted in a widespread depletion of myeloid cells. Focusing on sex‐specific effects, we re‐analyzed our data derived from short‐term (3 weeks) and long‐term (12 weeks) administration of PLX5622 and showed that more than 80% of myeloid cells in male mice become rapidly depleted. In contrast, the majority of myeloid cells in female mice were only depleted after 12 weeks of treatment, indicating a slower depletion rate in females. These findings were predominantly observed in WT mice, whereas both male and female transgenic animals show increased numbers of residual cells even after 12 weeks of treatment, an observation that has been described previously in APP‐PS1 mice (Unger et al. 2018). The observed sex difference might suggest that female myeloid cells may be more resilient toward CSF1R inhibition, or their depletion mechanism is different (Johnson et al. 2023). Accordingly, myeloid cell depletion efficiency was significantly higher in male C57BL6/J animals after 2 weeks (Le et al. 2025) and in a mouse model for neuronal ceroid lipofuscinoses after 5 months of PLX3397 administration (Berve et al. 2020). Contrarily, a study by Sharon et al. suggests that sex differences in depletion efficiency could be species‐dependent, as the authors describe a higher depletion efficiency in female Sprague Dawley rats after 10 days of PLX5622 treatment (Sharon et al. 2022).

Differences in depletion efficiency may also indicate pharmacological or pharmacokinetic differences across sexes, with males having higher plasma and brain concentrations of CSF1R inhibitors (Johnson et al. 2023), albeit similar consumption rates in males and females (Johnson et al. 2023; Le et al. 2025). Interestingly, Le et al. showed that the concentration of PLX3397 in the brain does not change significantly in females treated with a 1.4× higher dosage of PLX3397, whereas the concentration increased in males, suggesting a relevant change in uptake and/or metabolism in males and females (Le et al. 2025).

Transcriptional profiles of microglia differ between sexes (Villa et al. 2018), which may also be relevant in response to CSF1R‐mediated depletion. Although CSF1R expression on CD11b^+^CD45^int^‐sorted microglia did not differ statistically significantly between sexes, Le et al. observed a trend toward reduced CSF1R expression in females. Their data suggest distinct microglia subpopulations may be more responsive toward CSF1R‐mediated depletion (Le et al. 2025).

As discussed in our previous article (Battis et al. 2022), a thorough analysis of the myeloid subset composition in the brain of MSA patients and MBP29‐hα‐syn mice is still outstanding. CSF1R inhibitors have been suggested to impact peripheral immune subsets, including resident macrophages, circulating monocytes, and bone marrow–derived hematopoietic cells (Bosch et al. 2023; Claeys et al. 2023; Fliegauf et al. 2026; Lei et al. 2020), and therefore might impact potential infiltration of peripheral myeloid cells. On the other hand, recent studies have shown that monocytes are able to replace brain macrophages (including microglia) under certain circumstances such as breakdown of the blood–brain barrier (Mildner et al. 2007), repetitive PLX5622 treatment (Du et al. 2026), or sustained niche availability (Bastos et al. 2025). Whether any of these circumstances vary in a sex‐specific manner is still unknown, and further investigation is needed to determine the contribution of monocytes to the overall myeloid cell composition in male and female MSA patients and their respective preclinical models.

To evaluate the behavioral phenotype, MBP29‐hα‐syn mice were analyzed at 16 weeks of age when motor deficits are present (Shults et al. 2005). We observed no sex‐specific effects on the motor behavior of MBP29‐hα‐syn mice upon PLX5622 treatment, whereas the occurrence of dystonia‐like behavior was reduced in males. Sexual dimorphism has been described in adult‐onset idiopathic isolated focal dystonia, where women outnumber men for the majority of dystonia types (Kilic‐Berkmen et al. 2024; Rafee et al. 2021). Whether microglia play a role in dystonia pathomechanism and sex differences requires further investigation.

In our study, an increased depletion efficacy in male MBP29‐hα‐syn animals was accompanied by an increased length and density of TH^+^ fibers. An involvement of dopaminergic neurons in dystonia has been proposed (Ribot et al. 2019), suggesting that TH deficiency in females might explain the higher prevalence of dystonia‐like behavior.

In summary, male animals exhibited a rapid myeloid cell depletion upon PLX5622 administration, potentially preventing MSA‐related loss of TH^+^ fibers and subsequently delaying the onset of a disease‐specific phenotype, i.e., dystonia‐like behavior. Thus, we propose that sex‐specific modeling of therapeutic interventions must be preclinically considered and explored in MSA and other neurodegenerative diseases. Furthermore, sex‐specific depletion efficiency should be considered in future depletion experiments.

## Author Contributions

**Kristina Battis:** writing – original draft, visualization, investigation, formal analysis. **Michael Mante:** investigation. **Isabel Naumann:** writing – review and editing, investigation. **Marie Andert:** investigation, writing – review and editing. **Ha Yeon Kim:** investigation. **Wei Xiang:** resources, writing – review and editing. **Robert A. Rissman:** writing – review and editing, supervision, resources. **Jürgen Winkler:** funding acquisition, writing – review and editing, supervision, conceptualization, resources. **Alana Hoffmann:** conceptualization, investigation, funding acquisition, writing – original draft, visualization, methodology, supervision, formal analysis.

## Funding

The study was funded by the Deutsche Forschungsgemeinschaft (DFG, German Research Foundation) (270949263/GRK2162, 498972649, 505539112) and was supported by an Award from the Multiple System Atrophy Coalition. A.H. was funded by the *Erstantragssteller‐programm* of the IZKF (J92), BaCaTeC (Bavaria California Technology Center), and the DFG (498972649).

## Conflicts of Interest

The authors declare no conflicts of interest.

## Data Availability

The data supporting this study's findings are included in this published article. Further data are available in our initial study (Battis et al. 2022). Raw data are available from the corresponding author upon request.
